# Chaparral Shrub Hydraulic Traits, Size, and Life History Types Relate to Species Mortality during California’s Historic Drought of 2014

**DOI:** 10.1371/journal.pone.0159145

**Published:** 2016-07-08

**Authors:** Martin D. Venturas, Evan D. MacKinnon, Hannah L. Dario, Anna L. Jacobsen, R. Brandon Pratt, Stephen D. Davis

**Affiliations:** 1 Department of Biology, California State University, 9001 Stockdale Hwy, Bakersfield, CA 93311, United States of America; 2 Grupo de Investigación en Genética, Fisiología e Historia Forestal, Universidad Politécnica de Madrid, Avda. de las Moreras s/n, 28040, Madrid, Spain; 3 Natural Science Division, Pepperdine University, 24255 Pacific Coast Highway, Malibu, CA 90263, United States of America; Estacion Experimental de Zonas Aridas—CSIC, SPAIN

## Abstract

Chaparral is the most abundant vegetation type in California and current climate change models predict more frequent and severe droughts that could impact plant community structure. Understanding the factors related to species-specific drought mortality is essential to predict such changes. We predicted that life history type, hydraulic traits, and plant size would be related to the ability of species to survive drought. We evaluated the impact of these factors in a mature chaparral stand during the drought of 2014, which has been reported as the most severe in California in the last 1,200 years. We measured tissue water potential, native xylem specific conductivity, leaf specific conductivity, percentage loss in conductivity, and chlorophyll fluorescence for 11 species in February 2014, which was exceptionally dry following protracted drought. Mortality among the 11 dominant species ranged from 0 to 93%. Total stand density was reduced 63.4% and relative dominance of species shifted after the drought. Mortality was negatively correlated with water potential, native xylem specific conductivity, and chlorophyll fluorescence, but not with percent loss in hydraulic conductivity and leaf specific conductivity. The model that best explained mortality included species and plant size as main factors and indicated that larger plants had greater survival for 2 of the species. In general, species with greater resistance to water-stress induced cavitation showed greater mortality levels. Despite adult resprouters typically being more vulnerable to cavitation, results suggest that their more extensive root systems enable them to better access soil moisture and avoid harmful levels of dehydration. These results are consistent with the hypothesis that short-term high intensity droughts have the strongest effect on mature plants of shallow-rooted dehydration tolerant species, whereas deep-rooted dehydration avoiding species fare better in the short-term. Severe droughts can drive changes in chaparral structure as a result of the differential mortality among species.

## Introduction

Chaparral is the most abundant plant community in California and the shrubs that dominate this community are adapted to hot and dry summers and periodic fires [1]. Global-change type droughts (the combination of low precipitation and warmer temperatures) in recent years have led to woody plant die-offs in many areas across the globe (*e*.*g*., [2–7]). These large scale die-offs impact the structure, composition and dynamics of plant communities; impacts that consequently alter ecosystem function and threaten biodiversity and the wellbeing of humans [8]. This phenomenon may become more common in some areas where the climate is rapidly warming and drying. In California, for example, the climate has warmed by about 1°C in the last century and predictions are for continued warming and drying [9–11].

The physiological mechanisms that lead to these die-offs are not well established and this limits the ability to predict how ecosystems will respond to future drought events. Several hypotheses have been proposed relative to physiological mechanisms of drought mortality. High intensity droughts of short duration may cause abrupt mortality due to the breakdown of vascular (xylem) transport of water [12]. Alternatively, drought may lead to a slow decline in growth in the years following the drought and ultimately result in death [13]. Longer term droughts of lower intensity may be similar in leading to a reduction in growth and a decline in health of long-lived plants and their eventual death. Pests and pathogens are often a contributing factor in mortality of weakened plants [14,15]. Another hypothesis is that long-term droughts may cause some species to have a negative carbon balance and exhaust their carbohydrate stores leading to mortality [12], although the extent that carbon starvation may lead to mortality is controversial [16] as carbon is generally not the limiting factor under stress conditions [17]. All three factors, hydraulic failure, pathogen attack, and carbohydrate depletion, are non-mutually exclusive and may act together [18–21].

These factors do not affect all species similarly and an interesting observation is that some woody species have succumbed to recent droughts in large numbers while others have been less affected. An important question is why are some species more vulnerable to these events than others? A substantial number of studies have examined species differential drought tolerance from life history, functional and physiological trait perspectives (*e*.*g*., [20,22–26]). Nevertheless, many knowledge gaps remain, such as (i) species specific dehydration limits for survival, (ii) differences in mortality between the seedling establishment phase when rooting depths among species are equal, and the adult persistence phase when rooting depths among the same species are markedly varied, and (iii) relationships between survival strategies that utilize dehydration tolerance (low seasonal water potential and high cavitation resistance) versus dehydration avoidance strategies (deep roots that tap soil moisture resources imparting high seasonal water potential during seasonal drought). Further research is required in order to better predict the effects of increasing drought and climate change on plant community structure.

We examined the factors contributing to drought-associated mortality in a diverse chaparral shrub community of southern California. In this area, drought-associated mortality has already been observed during past droughts. Extensive branchlet dieback after a drought in 1995 was observed in the chaparral species *Ceanothus crassifolius* Torrey and was linked to hydraulic failure [27]. Postfire mortality of resprouting species of chaparral was observed during an intense drought in 2007 [24] and of mature chaparral plants in a stand located at a chaparral-desert ecotone in 2002 [28]. The severe California drought of 2014 offered the possibility to further examine traits associated with mortality in a diverse stand that contained more species than previously examined and during the most extreme drought to affect this region in the last 1,200 years [29,30]. Chaparral species life history types (LHT) can be classified into four different categories in relation to regeneration strategies after fire [31]: obligate seeders, those species that have to recruit from seeds (S+) and do not resprout (R-; R-S+); facultative seeders (R+S+), those that can establish from structures, such as lignotubers, that resprout after fire and also recruit from seeds; obligate resprouters (R+S-), those that only establish from resprouting structures after fire; and post-fire colonizers (R-S-), species that do not persist after fire and re-establish themselves by seed dispersal from unburnt populations. As LHT has been previously linked with differences in drought survival due to a tradeoff between adult persistence and seedling recruitment in water limited habitats [32,33], we predicted that mortality would be the greatest in obligate seeders and the lowest in obligate resprouters. We could not test LHT effects directly since the research site did not contain sufficient species with each LHT to include this as a factor in statistical analyses, but we were able to compare the observed species mortality patterns with previously published data. We hypothesized that a key mechanism causing mortality would be vascular failure during tissue dehydration and predicted that species would differ in this trait in conjunction with mortality [28], with the lowest hydraulic conductivities and the highest losses in conductivity in species that experienced the most mortality. Finally, we hypothesized that within a species plant size would be associated with drought survival because larger plants aboveground would also be larger belowground (due to the root:shoot biomass relationship) and this would allow them to avoid lethal levels of tissue dehydration during drought.

## Materials and Methods

### Ethics statement

All research was conducted in accordance with relevant national and international guidelines. Mountain Restoration Trust gave us permission to conduct the study in this site of their property. Field studies did not involve endangered or protected species.

### Study site

**Fig 1:**
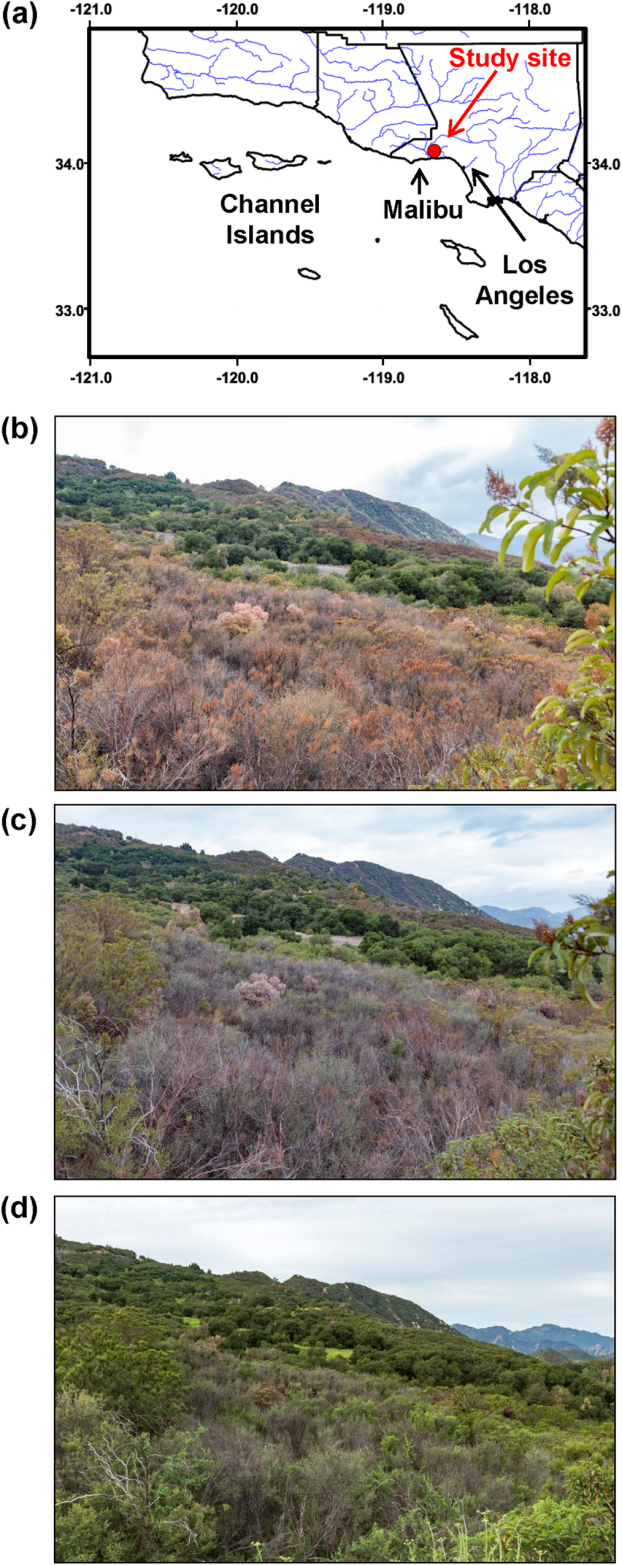
Location of the study site and images captured during the 2014 historic drought. (a) The study site was located in the Cold Creek Canyon, Santa Monica Mountains (California, USA) at 6.5 km from the coast. The grid is represented in degrees. (b) General view of the study site showing extensive dieback taken on March 2, 2014, (c) November 1, 2014, and (d) March 21, 2015, after winter rains. The *Malosma laurina* that can be observed at the forefront right (b and c) remains green, in contrast with the middle ground chaparral shrubs where plants where sampled, because it is presumed to have access to more water since it grows along the road experiencing less competition for soil moisture and enhanced runoff. At the back of the middle ground some riparian oaks grow along a stream and remain green. These images evidence the effects that the global change type drought is having on the upland vegetation. (Photographs: R. Brandon Pratt).

The study site was located in Cold Creek Canyon Preserve of the Santa Monica Mountains, California, USA (N 34° 05’, W 118° 39’), at about 350 m elevation and on a west-facing slope (Fig 1). The soil is derived from sandstone sedimentary rocks. The study site has a Mediterranean climate, characterized by a moist winter and a dry summer period of typically 5.5 months (S1 Fig). For the period 1998–2014 the mean annual temperature was 17.7°C and the mean annual rainfall 521.8 mm, whereas for the drought years 2012–2014 the mean annual temperature and rainfall were 18.3°C and 237.5 mm, respectively (Malibu Hills weather station; N 34° 03’, W 118° 38’, 480 m above sea level; USDA Forest Service; NESS ID: CA4173E; 3.86 km from the study site). The combination of lower precipitation and warmer temperatures is consistent with a global-change-type drought [3,34].

The stand is composed of a 21-year-old, mature mixed chaparral community that contains over 11 species of shrubs (Table 1). These species can be classified in three different LHTs in relation to their regeneration strategy after fire (Table 1): obligate seeder (R-S+), facultative seeder (R+S+), and obligate resprouter (R+S-). The stand last burned on November 2, 1993; at that time the stand was 23 years old with the previous fire occurring in 1970 [35]. For southern California, the average fire return period for chaparral stands is of 30–40 years, which is estimated to be more frequent than pre-European colonization due to the direct relationship existing among fire frequency and population density [36]. The average between-fire interval for all natural areas in the Santa Monica Mountains from 1925 to 2001 was 32 years [37].

**Table 1:** Species analysed in this study and their life history traits and maximum vessel length.

Code	Scientific name	Familiy	Common name	Fire life history type^†^	Maximum vessel length (m)^‡^
Af	*Adenostoma fasciculatum* Hook. & Arn.	Rosaceae	Chamise	R+S+	0.29
As	*Adenostoma sparsifolium* Torr.	Rosaceae	Red shank	R+S+	0.48
Ag	*Arctostaphylos glauca* Lindley	Ericaceae	Big berry manzanita	R-S+	0.30
Cc	*Ceanothus cuneatus* Nutt.	Rhamnaceae	Buck brush	R-S+	0.23
Cs	*Ceanothus spinosus* Nutt.	Rhamnaceae	Greenbark ceanothus	R+S+	0.30
Cb	*Cercocarpus betuloides* Torrey & A. Gray	Rosaceae	Mountain mahogany	R+S-	1.04
Ha	*Heteromeles arbutifolia* (Lindl.) M. Roem.	Rosaceae	Christmas berry	R+S-	0.86
Ml	*Malosma laurina* (Nutt.) Abrams	Anacardiaceae	Laurel sumac	R+S+	0.92
Qb	*Quercus berberidifolia* Liebm.	Fagaceae	Scrub oak	R+S-	1.60
Ri	*Rhamnus ilicifolia* Kellogg	Rhamnaceae	Hollyleaf redberry	R+S-	1.23
Ro	*Rhus ovata* S. Watson	Anacardiaceae	Sugar bush	R+S+	1.40

^†^ R-S+, obligate seeder; R+S+, facultative seeder; and R+S-, obligate resprouter.

^‡^ Data from [38,39].

### Site characterization and mortality evaluation

A point-quarter sampling (PQS) technique was employed in February-March 2014 to characterise the study site, species composition, and structure [40,41]. Sixty-three points were randomly selected throughout the stand. The area around each point was divided into four equal sectors (quarters) based on compass readings. The individual nearest to the sampling point within each sector was identified, its species determined, and the distance from the point to the centre of the rooted base of the plant measured. Plant height was measured and the basal and crown diameters determined with two perpendicular measurements. Each plant was also categorized as ‘dead’ or ‘alive’ to determine mortality and infer community shifts. A plant was determined dead when the canopy was totally dry and brittle and it had no green leaves and no green bark. Therefore, we were conservative determining death rate and if a plant had 99% of canopy dieback and still had one small green branch we classified it as alive. When plants were measured in the point quarter sampling we only took into consideration those that were alive or had died recently (<1 year). This was discernible because recently deceased plants had dried leaves attached to stems and intact bark. We omitted measuring plants that were dead for a longer period, such as those that had died in the previous 2006 drought. These were clearly “old dead” plants and contained no retained leaves and often had shed all of their bark. After the onset of March-May 2015 rains, the site was revisited and tagged plants re-evaluated. In all cases, plants that had been categorized as “dead” remained dead, and those considered “alive”, remained alive.

A systematic sampling (SYS) was also performed in March 2014 for characterizing more accurately the mortality percentage of those less frequent species from which few (*i*.*e*. *C*. *spinosus*, *M*. *laurina*, *R*. *ilicifolia*, and *R*. *ovata*) or no individuals (*i*.*e*. *C*. *betuloides*, *H*. *arbutifolia*, and *Q*. *berberidifolia*) were characterized with PQS (Table 2). The site was searched in order to locate as many individuals as possible of these species and characterize their status (dead or live).

**Table 2:** Mortality estimated by point quarter sampling and systematic sampling in February-March 2014.

	Point quarter sampling*			Systematic sampling		
Species	# of sampled plants	# of dead plants	Mortality (%)	# of sampled plants	# of dead plants	Mortality (%)
*A*. *fasciculatum*	86	54	62.8			
*A*. *glauca*	45	35	77.8			
*A*. *sparsifolium*	23	2	8.7			
*C*. *betuloides*				6	0	0.0
*C*. *cuneatus*	77	55	71.4			
*C*. *spinosus*	12	11	91.7	150	140	93.3
*H*. *arbutifolia*				8	1	12.5
*M*. *laurina*	4	0	0.0	28	1	3.6
*Q*. *berberidifolia*				28	1	3.6
*R*. *ilicifolia*	3	3	100.0	21	7	33.3
*R*. *ovata*	2	0	0.0	33	0	0.0
**Total**	**252**	**160**	**63.5**	**274**	**150**	**54.7**

Note that 3 of the studied species were not detected with PQS.

* Data from 63 sampling points.

The 46 permanent quadrats of 1 m^2^ established immediately after the 1993 wildfire for determining *A*. *fasciculatum* and *A*. *sparsifolium* seedling emergence and their long term survival [35,42] were also evaluated in March 2014. In these quadrats, seedling emergence and survival were also evaluated for *C*. *cuneatus*. Immediately after the 1993 fire, 100 lignotubers from both *Adenostoma* species were randomly selected, without knowing if they were dead or alive, and labelled with wire and metal tags for evaluating the percentage that survived the fire (successfully resprouted) and to monitor their postfire survival and growth through time. In March 2014, as many of these tagged plants as possible were located (86 *A*. *sparsifolium* and 70 *A*. *fasciculatum*) and their status characterized (dead or live). In addition, mortality was also evaluated in another 9 permanent quadrats of 1 m^2^ that had been established in January 1994 under *A*. *glauca* burnt plants in order to evaluate the postfire seedling emergence and survival of this species.

### Hydraulic traits and physiological mechanisms associated with mortality

Hydraulic traits from 11 species (Table 1) growing at the study site were evaluated. On February 24–26, 2014, when the study site had only received 51.6 ± 5.1 mm of precipitation in the previous 12 months (mean ± SE calculated from the three closest weather stations; S2 Fig), 6–12 large branches (> 2 m length) per species were collected at predawn. The branches were cut in air and only one branch was collected per plant. The branches were immediately placed in opaque double-plastic-bags containing a piece of wet tissue paper to minimize post-cutting dehydration. They were transported to a laboratory 2 h away at California State University, Bakersfield. The bags were placed in a dark room at 15°C and covered with a wet sheet to minimize water loss though the plastic until samples were measured. All hydraulic measurements were performed the same day that the samples were collected.

Xylem water potential (Ψ_pd_) was measured on three side branchlets of each large branch. Care was taken to not sample from the main stem from where the segment being targeted for hydraulic measurements was located in order to not induce cavitation. The branchlets were collected immediately after opening the large double bags, placed individually in zip-bags, and stored in a cool chamber until they were measured (<1h) with a pressure chamber (Model 2000, PMS Instrument Co., Albany, OR, USA).

Hydraulic conductivity was evaluated on one 14 cm stem segment per large branch. The diameter with bark of these segments was 4.7–8.7 mm. Out of precaution not to artificially induce embolism, the tension of the branches was relaxed prior to excising the segment on which measurements were performed [43]. This was done by progressively cutting under water 10–20 cm off from the base of the branch every 20–30 s, until the selected segment located at a distance greater than 1× the maximum vessel length (Table 1) from the first cut was reached. This procedure was repeated from the distal end of the segment until a 15–17 cm long segment was excised. Both ends of the segments were trimmed under water with a new razor blade to 14 cm. Initial stem hydraulic conductivity (K_h_) was measured gravimetrically with a stem conductivity apparatus [44]. This system measures the water flow through a stem by connecting it with tubes to two reservoirs located at different heights, creating a pressure gradient that drives flow. The lower reservoir is placed on an analytical balance (CP124S, Sartorius, Goettingen, Germany). K_h_ is calculated as the quotient between the mass flow rate and the pressure gradient, multiplied by stem length [44]. Measurements were performed using a low pressure head (1.5–2.0 kPa) in order to avoid displacing native emboli. In order to increase accuracy, K_h_ was corrected for negative background flow that may occur when dehydrated tissues absorb water when the pressure head is 0 kPa [45]. Measurements were performed with a 20 mM KCl degassed (membrane contactor, Liqui-Cel Minimodule 1.7×5.5, Charlotte, NC, USA) and filtered (0.1 μm inline filter, GE Water and Process Technologies, Trevose, PA, USA) solution. Following initial K_h_, stem segments were flushed for 1 hour at 100 kPa with the 20 mM KCl degassed solution to remove all emboli, and maximum conductivity (K_max_) measured. The native percentage loss of conductivity (PLC) of each stem was calculated as PLC = (1 –K_h_ / K_max_) × 100. Xylem native specific conductivity (K_s_) was calculated dividing K_h_ by sapwood cross-section area of the distal end of the segment. All live or barely live leaves located distally from the measured segment were collected and their area (A_L_) measured with a leaf area meter (Li-3100C, LI-COR, Lincoln, Nebraska, USA). Native leaf specific conductivity (K_L_) was calculated as K_h_ divided A_L_.

In order to evaluate the effect of tissue dehydration on leaf function, the water potential at midday (Ψ_md_) and the maximum quantum efficiency of photosystem II (F_v_/F_m_) of the studied species were evaluated on February 14–15, 2014. Fluorescence was measured using a pulse-modulated fluorometer (Opti-Sciences, OS1-FL, Tyngsboro, Massachusetts, USA). When light is absorbed by the light harvesting complex in the thylakoid membranes of chloroplasts, the resulting energy in the form of excited electrons can drive photosynthesis or be dissipated as heat or fluorescence. These processes are competitive, thus, a fluorometer evaluates the amount of energy that the chloroplasts are able to use in the light reactions of photosynthesis. Dark adapted fluorescence has been shown to be proportional to maximum quantum efficiency of photosystem II [46]. Values of 0.8–0.83 indicate maximum quantum efficiency for dark adapted leaves and values below 0.8 indicate varying degrees of photoinhibition. Fluorescence was measured on dark adapted leaves that were covered for 30 min with leaf clips. These measurements were also performed on March 21, 2015, when surviving plants had rehydrated after winter rains (S2 Fig).

Predawn (Ψ_pd_) and midday (Ψ_md_) water potentials of the 11 chaparral species were also evaluated at the end of the 2014 summer dry period (October 26). It had not rained at the site since April 2014, and 2014 winter-spring rainfall was significantly below average totalling only 111.4 ± 11.8 mm (mean ± SE, n = 3; S2 Fig). One branchlet was collected from each plant (6–9 plants per species), placed individually in zip-bags, and stored in a cool-box with ice-packs until they were measured (<3h) with a pressure chamber.

### Data analyses

PQS data were analysed using standard methods [41]. We calculated the average basal area (m^2^), average crown area (m^2^), density (plants ha^-1^), relative density (%), relative frequency (%), and relative dominance (%) based on basal area and crown area of each species (S1 Appendix). These parameters were calculated from the data of both live and dead plants, and therefore, are representative of the stand structure prior to the 2014 drought. Multiplying the total number of plants by the species specific survival ratio also allowed us to estimate the relative density and dominance after the drought.

The PQS data were analysed with Generalized Linear Models (GLMs) in order to determine the factors involved in plant mortality. Models were evaluated with JMP 9.0.0 (SAS Institute Inc., NC, USA). Species with 4 or less observations in the PQS were not included in the survival GLMs (*i*.*e*. *M*. *laurina*, *R*. *ilicifolia*, and *R*. *ovata* were excluded; Table 2) nor were the three species that were not detected by the PQS due to their low frequency (*i*.*e*. *C*. *betuloides*, *H*. *arbutifolia*, and *Q*. *berberidifolia*). Thus, data from 243 individuals belonging to the five most frequent species in the stand (*i*.*e*. *A*. *fasciculatum*, *A*. *sparsifolium*, *A*. *glauca*, *C*. *cuneatus*, and *C*. *spinosus*) was used for constructing GLMs. The response variable was binary (live = 1; dead = 0). These models were constructed with the *logit* link function (one of JMP link function options) and maximum likelihood estimation method. The factors evaluated were species (Sp), plant height (H), basal area (BA), crown area (CA) and local density (D). Several models including different combinations of these factors were evaluated (S1 Table). The Chi Square Test was performed to determine the significance of the models. The Akaike Information Criterion (AIC) was used to select the best fitting model, *i*.*e*. the one with the lowest AIC value.

Hydraulic and physiological traits differences among species were evaluated with a one-way analysis of variance (ANOVA). Within a species, seasonal differences in Ψ_pd_ were evaluated with an ANOVA. The relationship between variables was studied with Pearson product-moment correlation. In order to select the function that best fitted the data, data were fitted to linear, exponential, second grade polynomic, and logarithmic functions with the *nls* command in R version 3.0.2 [47]. The AIC was used to select the best fit model. The comparison of K_s_ measured in this study with values obtained for these species for the same site from the literature [48] of different years and seasons was performed with a two-sample *t*-test.

## Results

**Fig 2:**
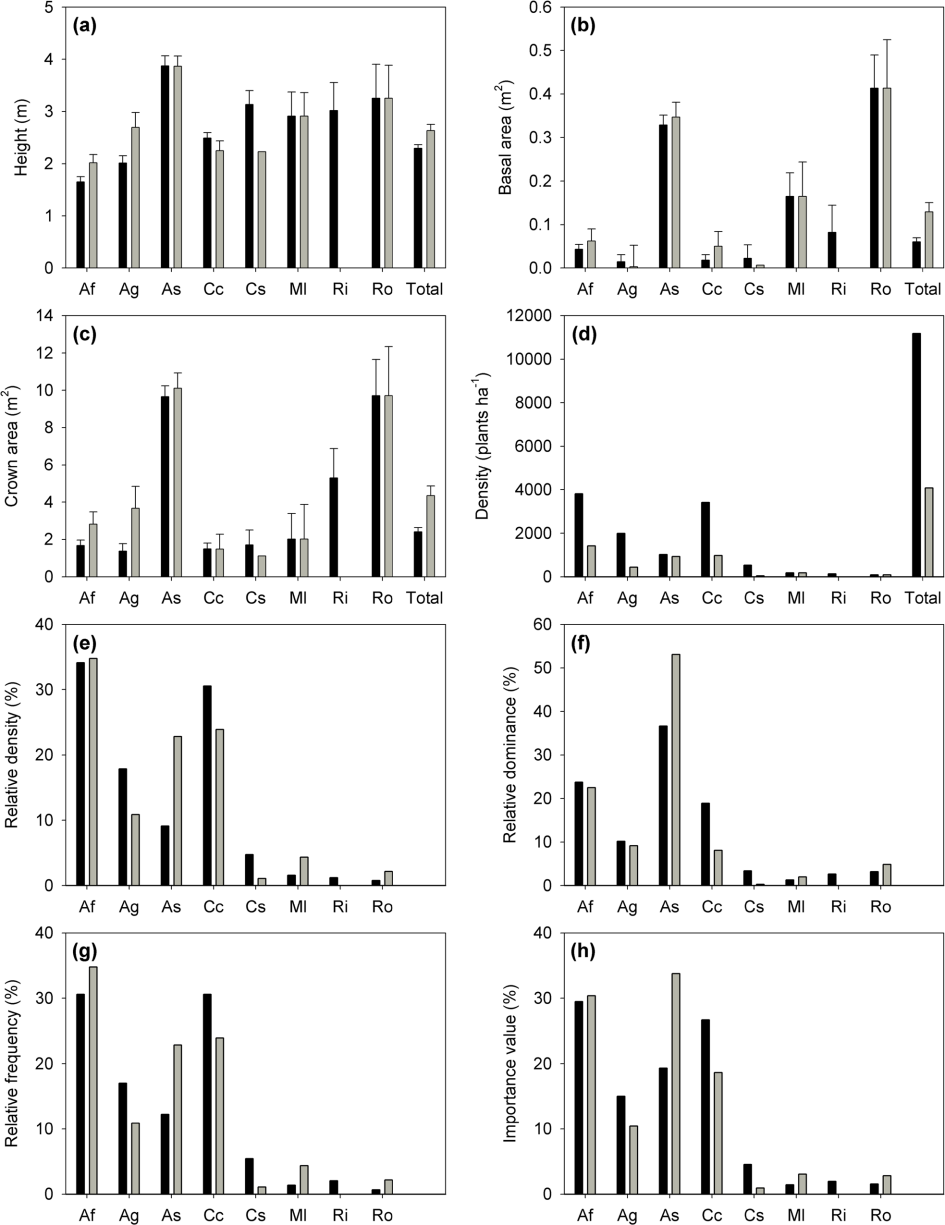
Stand structure prior to (black bars; all plants) and after (grey bars; only live plants) the 2014 drought inferred with point quarter sampling (PQS). Stand structure is represented as species (a) plant height, (b) basal area, (c) crown area, (d) plant density, (e) relative density, (f) relative dominance, (g) relative density, and (h) importance value. Species codes are reported in Table 1. Error bars represent standard error.

The stand had a density of 11,171 plants ha^-1^ (Fig 2D). Shrubs had a mean height of 2.29 ± 0.07 m (±SE), mean basal area of 0.061 ± 0.009 m^2^ (±SE), and mean crown area of 2.41 ± 0.23 m^2^ (±SE) (Fig 2A, 2B and 2C). The species with largest plants were the postfire resprouting species *A*. *sparsifolium* and *R*. *ovata* (Fig 2A, 2B and 2C). The species that showed higher density, frequency, and dominance, ordered from higher to lower importance value, were *A*. *fasciculatum*, *C*. *cuneatus*, *A*. *sparsifolium*, and *A*. *glauca* (Fig 2E, 2F, 2G and 2H). Three of the species that are obligate resprouters, *C*. *betuloides*, *H*. *arbutifolia*, and *Q*. *berberidifolia*, were not detected with PQS due to their low frequency and density (Table 2).

Mortality among the 11 species ranged from 0% (*C*. *betuloides* and *R*. *ovata*) to 93% (*C*. *spinosus*; Table 2). In those species where mortality was calculated by both PQS and SYS, the results below refer to SYS data only because these results are more accurate due to a larger sample size. Mortality reduced total stand density 63.4% (Fig 2D). Stand structure was modified and relative importance of species varied due to the differential mortality among species. After the 2014 drought, *A*. *sparsifolium* gained and *A*. *fasciculatum* maintained relative importance, whereas relative importance was reduced for *A*. *glauca*, *C*. *cuneatus*, and *C*. *spinosus* (Fig 2H).

**Fig 3:**
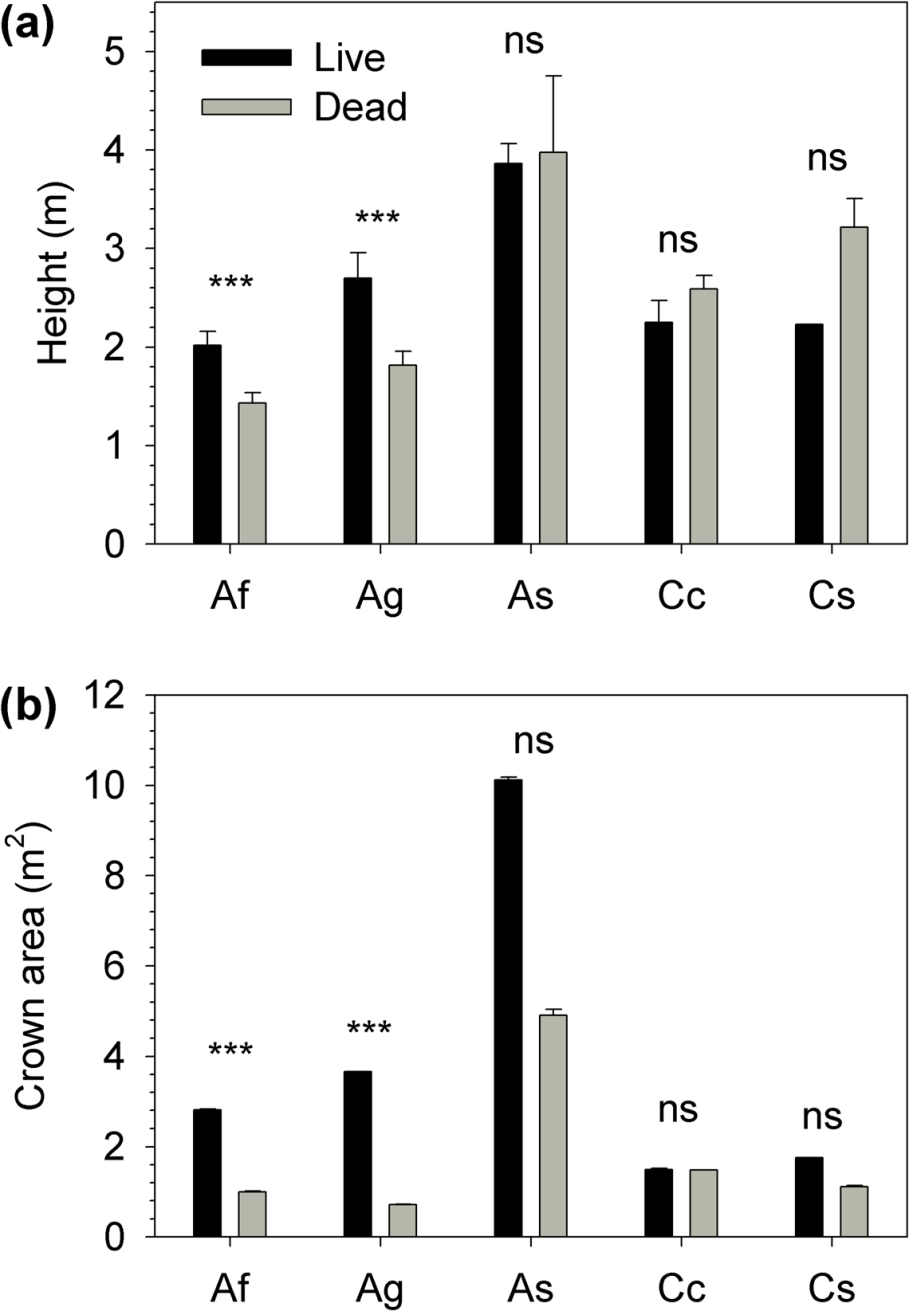
**Mean plant height (a) and crown area (b) of plants of the species analysed with GLMs that survived (black) and died (grey) during the drought.** Codes of the species are reported in Table 1. Error bars represent standard error. Significance levels of a two-side t-test: P > 0.05, ns; P < 0.05, *; P < 0.01, **; P < 0.001, ***.

The GLM that best explained mortality had species and crown area nested within species as main factors (AIC = 260; Table 3 and S1 Table). *A*. *sparsifolium* showed significant differences in the species parameter (S2 Table), because it had the lowest mortality of the five species analysed in the model (8.7% *vs*. > 62.8%; Table 2). Crown area nested within species was a significant effect (Table 3) because plants of *A*. *fasciculatum* and *A*. *glauca* with larger crowns had greater survival (Fig 3). The result was an increase in mean crown area and plant height for these species after the drought of 2014 (Fig 2A and 2C).

**Table 3:** Mortality GLM table for (a) whole model and (b) effect tests.

**(a) Whole model test**		** **	** **	** **
**Model**	**-Log Likelihood**	**L-R Chi Square**	**DF**	**Prob. > Chi Square**
Difference	38.36	76.72	9	**< 0.0001**
Full	119.55			
Reduced	157.91			
**(b) Effect Tests**				
**Source**		**L-R Chi Square**	**DF**	**Prob. > Chi Square**
Species		12.32	4	**0.0151**
Crown Area [Species]		34.72	5	**< 0.0001**

**Fig 4:**
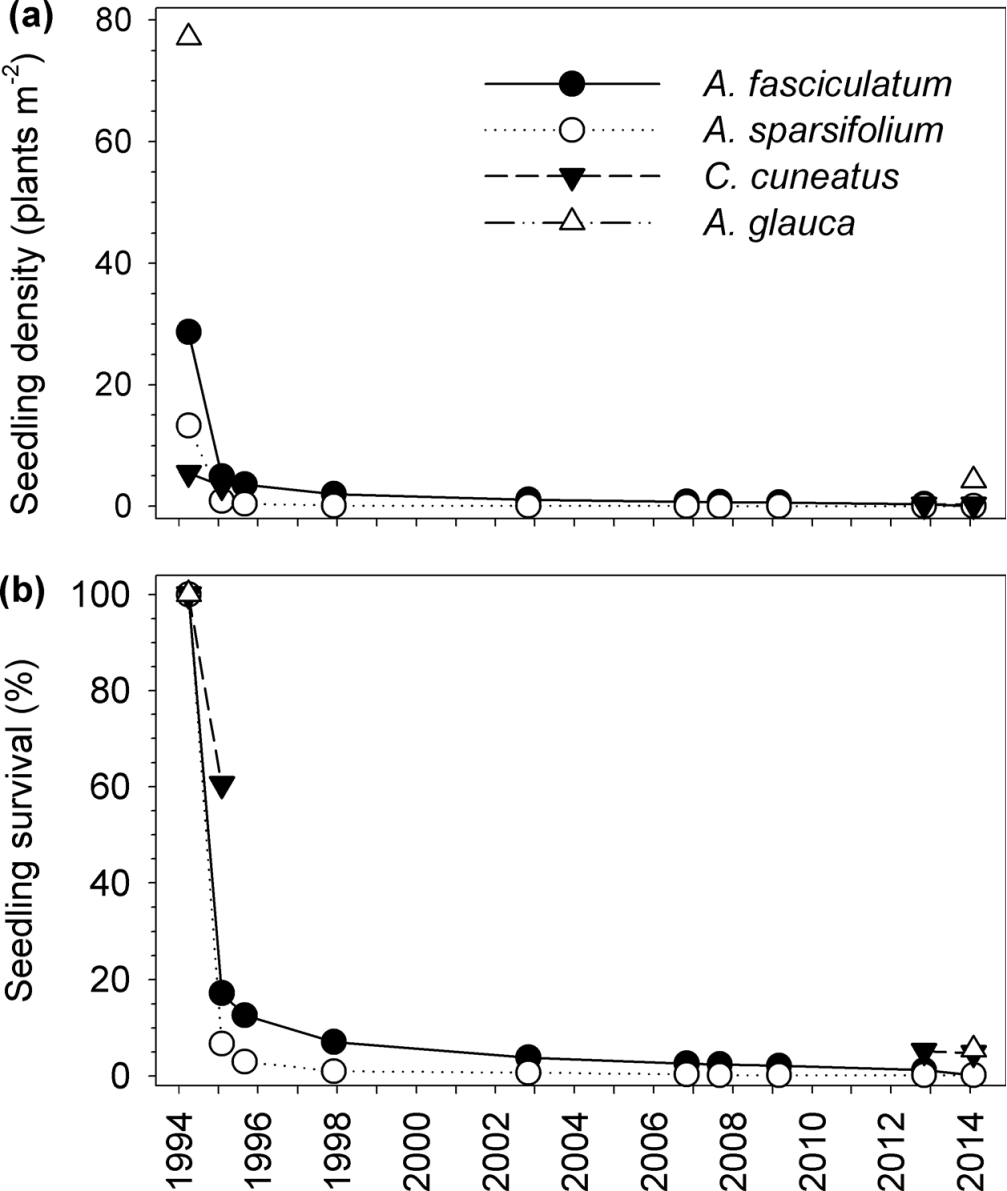
Seedling emergence after 1993 wildfire and survival over the years evaluated from the permanent quadrats. Expressed as (a) live seedlings per square metre and (b) percentage of live seedlings in relation to the ones that emerged. Data from 46 permanent quadrats (each 1 m^2^) for *A*. *fasciculatum*, *A*. *sparsifolium*, and *C*. *cuneatus*, and 9 permanent quadrats (each 1 m^2^) for *A*. *glauca*. *Adenostoma* survival data from 1993 to 2008 was previously published by [42].

The long term survey on permanent quadrats established after 1993 fire at the site showed that very few seedlings that emerged were still alive after 2014 drought (Fig 4A), with most mortality having occurred shortly after the fire among juvenile plants. In the case of *A*. *fasciculatum* and *A*. *sparsifolium* only 0.15% (2 out of a total of 1,319 plants) and 0.16% (1 out of a total of 610 plants), respectively, remained alive in 2014 (Fig 4B). Survival was slightly higher for *C*. *cuneatus* (4.74%) and *A*. *glauca* (5.48%; Fig 4B). Final densities (Fig 4A) of these quadrats are consistent with total species density in the study site (Fig 2D) for all species except *A*. *glauca*. The density of the latter was overestimated in quadrats because these were not placed randomly; they were placed under big *A*. *glauca* shrubs in order to have enough seedlings on which to evaluate long term survival.

The *Adenostoma* lignotubers tagged after 1993 wildfire revealed that all survived the fire, as 100% of them resprouted in 1994. The mortality for *A*. *sparsifolium* was 0% in 2002 (all 100 tagged plants were located) and 1.2% in 2014 (86 plants were located) and for *A*. *fasciculatum* 2.0% in 2002 (99 plants were located) and 25.7% in 2014 (70 plants located). *A*. *sparsifolium* 2014 mortality of tagged plants was slightly lower than the calculated by PQS (1.2% vs. 8.7%), whereas *A*. *fasciculatum* mortality of tagged plants was significantly lower than the estimated by PQS (25.7% vs. 62.8%).

**Fig 5:**
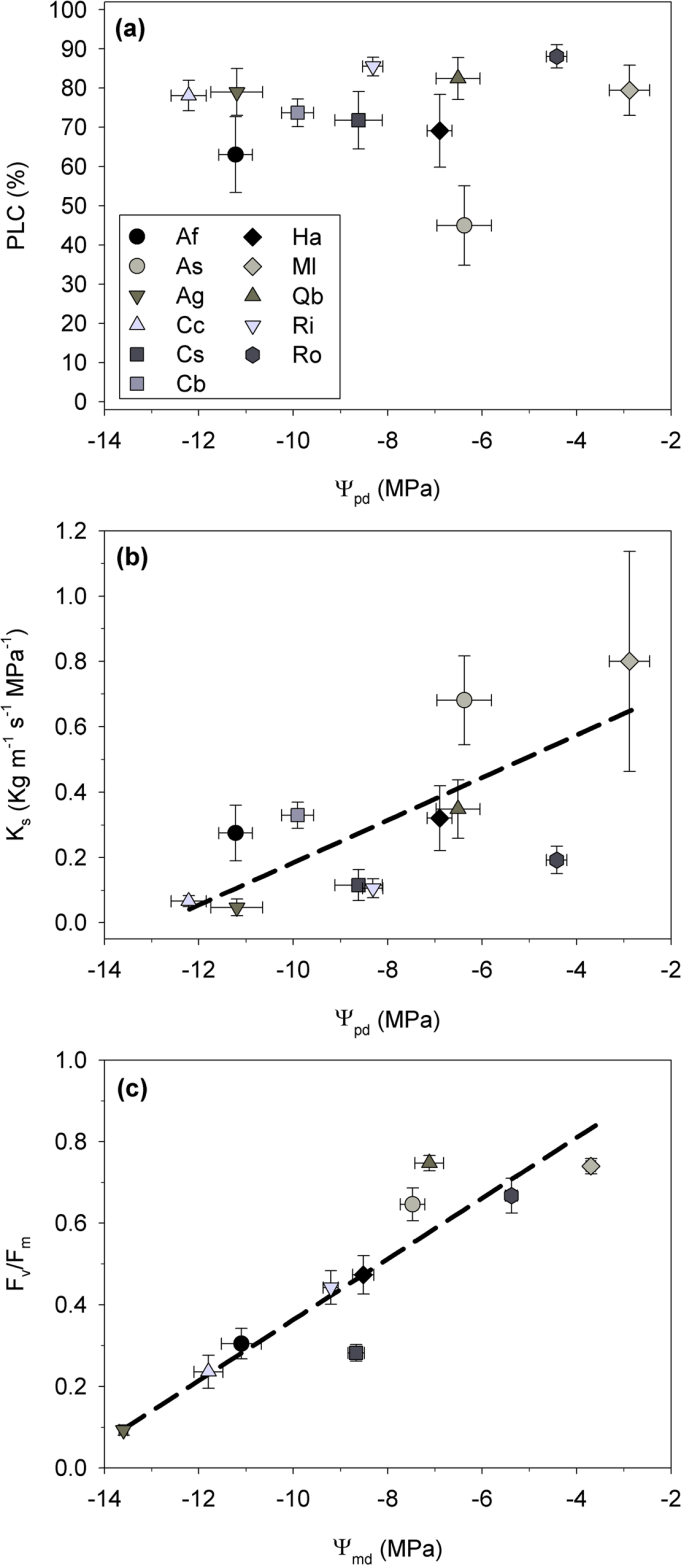
Correlations among water potentials, hydraulic parameters and fluorescence measured in February 2014. (a) Xylem water potential at predawn (Ψ_pd_) *vs*. percent loss in hydraulic conductivity (PLC) (means ± SE, n = 6–12); (b) Ψ_pd_ *vs*. xylem specific conductivity (K_s_) (means ± SE, n = 6–12); (c) midday xylem water potential (Ψ_md_) *vs*. dark adapted fluorescence (F_v_/F_m_) (means ± SE, n = 7–15). Broken line represents significant correlations (P < 0.05) among variables. Codes of the species are reported in Table 1.

Species showed significant differences in Ψ_pd_ (F_(10,80)_ = 36.138; P < 0.001), PLC (F_(10,80)_ = 2.749; P = 0.006), K_s_ (F_(10,80)_ = 4.774; P < 0.001), Ψ_md_ (F_(9,97)_ = 126.39; P < 0.0001) and F_v_/F_m_ (F_(9,78)_ = 44.238; P < 0.0001) at the peak of 2014 drought (Fig 5). The species with highest Ψ_pd_ were *M*. *laurina* and *R*. *ovata*, whereas the ones with lowest Ψ_pd_ were *C*. *cuneatus*, *A*. *glauca*, and *A*. *fasciculatum*. This same pattern among species was observed for Ψ_md_. All species showed high levels of native embolism (PLC > 60%; Fig 5A), except *A*. *sparsifolium* which had the lowest PLC (44.9%). The highest K_s_ values were recorded for *M*. *laurina* and *A*. *sparsifolium*, and the lowest for *C*. *cuneatus* and *A*. *glauca* (Fig 5B). *Q*. *berberidifolia* and *M*. *laurina* showed the highest F_v_/F_m_ and *A*. *glauca* the lowest (Fig 5C). Ψ_pd_ was correlated with K_s_ (n = 11, r = 0.67, P < 0.05; Fig 5B) but not with PLC (n = 11, P > 0.05; Fig 5A). Ψ_md_ and F_v_/F_m_ were correlated (n = 10, r = 0.91, P < 0.05; Fig 5C).

**Fig 6:**
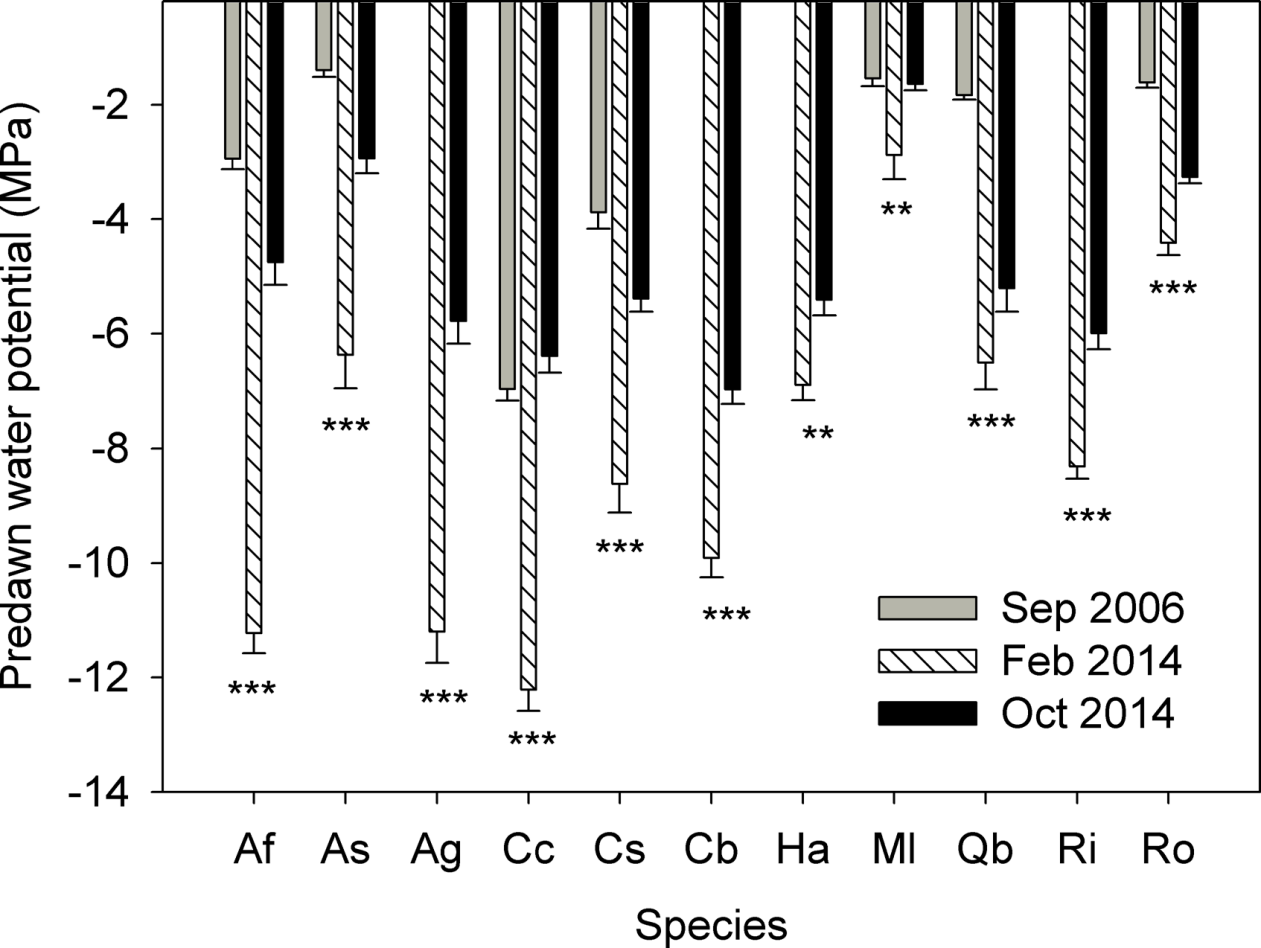
Comparison among predawn water potentials (Ψ_pd_) during 2006 (data from [48]) and 2014 droughts. Bars are means ± SE, n = 6–12. ANOVA significance levels comparing each season’s Ψ_pd_ within each species: P > 0.05, ns; P < 0.05, *; P < 0.01, **; P < 0.001, ***.

**Fig 7:**
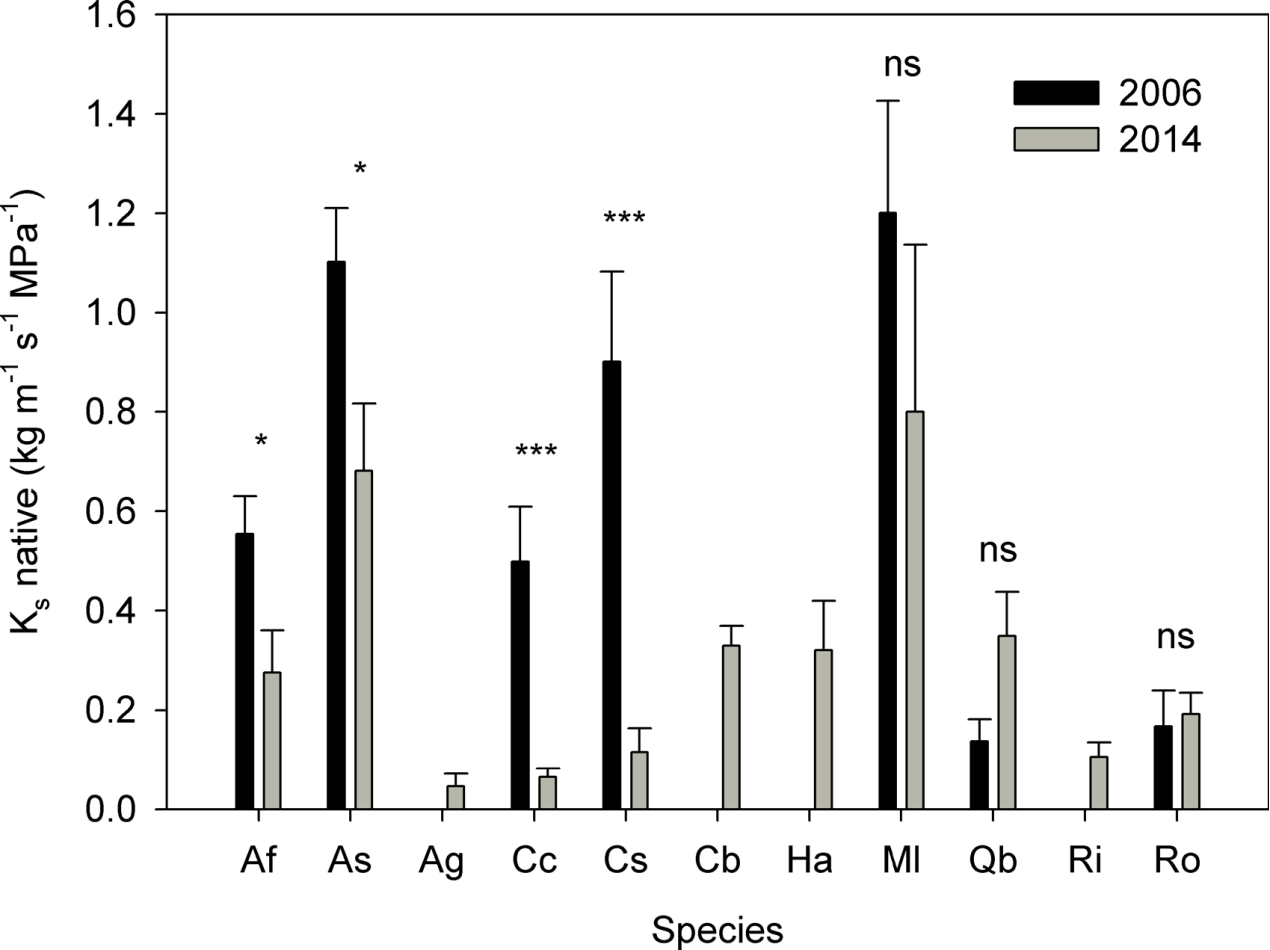
Comparison of native xylem specific conductivity (K_s_) of the studied species during the 2006 drought (data from May 2006; [48]) and 2014 drought (February 2014). Bars are means ± SE, n = 5–12. Significance levels for the double side t-test comparing both years: P > 0.10, ns; P < 0.10, *; P < 0.05, **; P < 0.01, ***.

All species were more dehydrated in February 2014 than in the previous extreme drought (September 2006) and later in the season (October 2014; Fig 6). Plants that survived the 2014 drought had rehydrated after 2015 winter rains and the mean Ψ_md_ of all species was higher than -2.5 MPa (March 2015, n = 6–7). Native K_s_ was lower in 2014 compared to 2006 drought for 4 species (Fig 7). Surviving plants quantum efficiency recovered with winter rains (mean F_v_/F_m_ ranged between 0.76 and 0.82 for all species in March 2015, n = 6–7).

**Fig 8:**
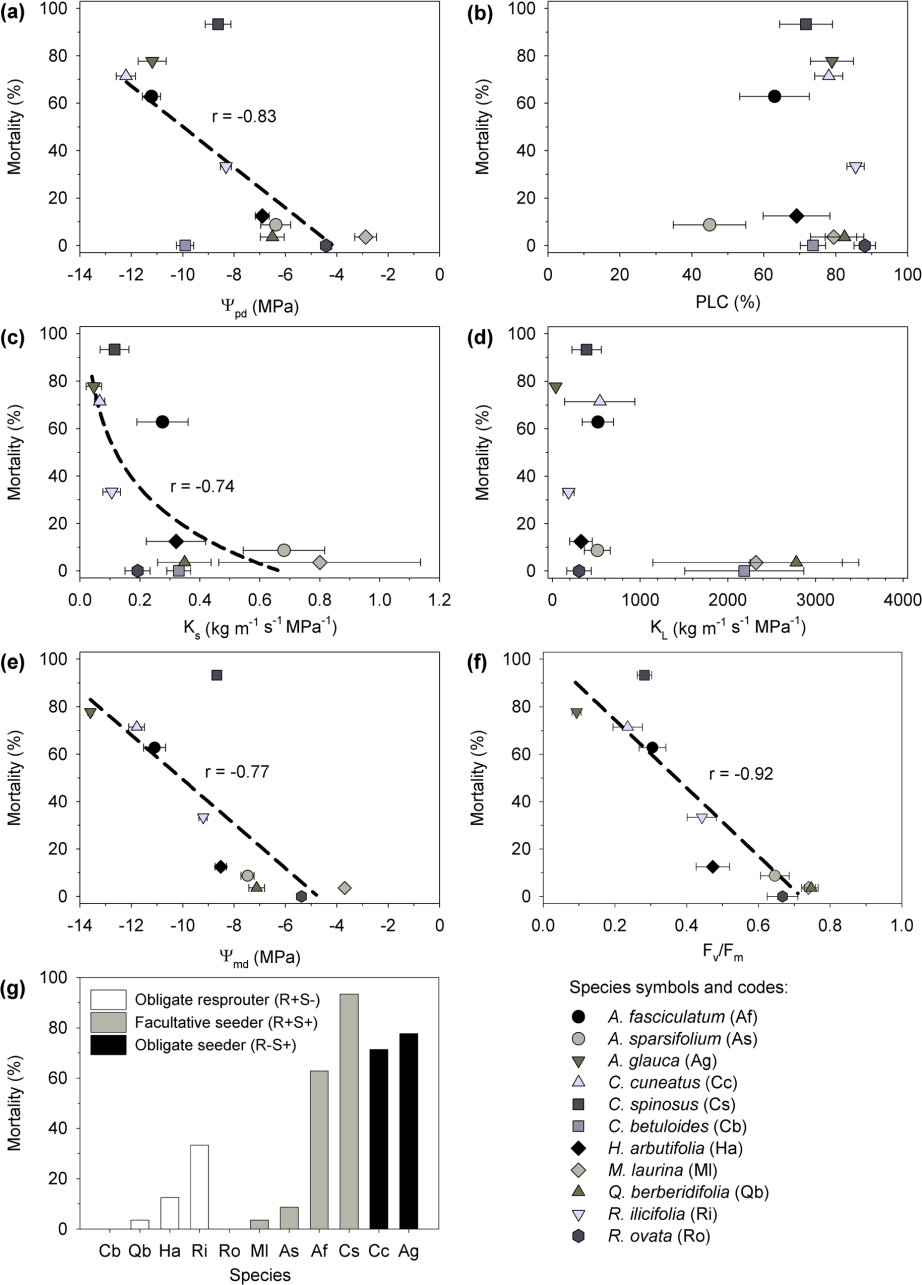
Relationship between mortality and hydraulic traits and life history types. Relation between mortality and (a) predawn water potential, Ψ_pd_, (b) percent loss in hydraulic conductivity, PLC, (c) xylem specific hydraulic conductivity, K_s_, (d) leaf specific hydraulic conductivity, K_L_, (e) midday water potential, Ψ_md_, (f) dark adapted fluorescence, F_v_/F_m_, and (g) life history type. Broken lines represent significant correlations (P < 0.05) among variables (K_s_ was log-transformed for the correlation analysis).

Mortality was negatively correlated with Ψ_pd_, K_s_ (log-transformed), Ψ_md_ and F_v_/F_m_ (P < 0.05; Fig 8A, 8C, 8E and 8F), whereas PLC and K_L_ were not correlated with mortality (P > 0.05; Fig 8B and 8D). It is to be noted that mortality and physiological measurements were not performed on the same individuals. These statistical tests were performed on averaged values for the different species. The species that showed higher mortality were obligate seeders and facultative seeders (Fig 8G).

## Discussion

Our data supports the hypotheses that life history type, hydraulic traits, and plant size were related to differential drought survival of chaparral shrubs during a high intensity climate-change type drought. These results also indicate that drought episodes, such as the one that affected California in 2014, may have severe effects on ecosystems and can alter species relative importance and dominance in chaparral communities. This will have implications for ecosystem function for numerous reason that include alterations in stand structure (more open), hydrology (*e*.*g*. more precipitation will directly hit the ground), nutrient cycling over the short and long-term (the *Ceanothus* spp. are nitrogen fixers and have been greatly reduced in numbers), openings in the canopy will make the community more invasible, and the dead biomass means that the site will likely burn at a higher intensity when the next fire comes.

Chaparral life history types have been shown to correlate with functional strategies and traits related to water stress resistance [23,49–51]. Previous studies have determined that obligate seeders (R-S+) have greater resistance to water-stress induced cavitation of stem and root xylem than facultative seeders (R+S+) and obligate resprouters (R+S-) [23,31,38,49,51], which is indicative of R-S+ species having a dehydration tolerance strategy whereas R+S+ and R+S- are closer to the dehydration avoidance end of the spectrum. In our study, the two R-S+ species (*A*. *glauca* and *C*. *cuneatus*) showed greatest tissue dehydration (Fig 8A and 8E), whereas the most hydrated species (*M*. *laurina* and *R*. *ovata*) were R+S+. This is consistent with R-S+ seedlings recruiting after fire in arid and exposed microsites with strong selection pressure for tolerating unavoidable tissue dehydration and high light and heat stress [51–53].

However, the greater cavitation resistance of R-S+ did not result in greater survival during high intensity drought conditions. In our study, R-S+ species had greater mortality than R+S- despite their xylem being more resistant to water-stress induced cavitation. This is consistent with previous chaparral drought mortality studies [23,28,54,55]. More specifically, we found the same mortality pattern as [28] observed during the 2002 drought in Southern California for the four species that were analysed in both studies (*i*.*e*. *A*. *glauca*, *A*. *fasciculatum*, *A*. *sparsifolium* and *R*. *ovata*). Resprouters (R+S+ and R+S-) that survive fire have more time than R-S+ to accumulate below ground biomass, therefore they usually have more extensive and deeper (in some cases) root systems, which enables greater access to moisture and mitigates drought effects, ultimately increasing their survival during a high intensity drought [28,31,56]. Greater canopy dieback and mortality during intense drought has also been observed for tree species with higher dehydration tolerance and shallow-root systems in Australia and North America [2,57]. Therefore, these observations in chaparral species are consistent with the hypothesis that high intensity droughts generate higher mortality among species that are more tolerant to tissue dehydration [24,28]. Nevertheless, this pattern is not followed by some species like *C*. *betuloides* that has a shallow root system [58], high tolerance to dehydration [38], and low drought mortality (Table 2).

The importance of rooting patterns and dehydration tolerance may vary in other life stages, such as during recruitment. Shrub and tree seedlings have a positive relationship between rooting depth and drought survival (*e*.*g*., [59,60]). In chaparral, when rooting depths are equal during seeding establishment, mortality is related to dehydration tolerance [23,52,61]. Therefore, dehydration tolerance may be a successful strategy for normal summer-drought conditions and during colonization after a disturbance, but may not confer higher survival in extreme drought events. This results in differential patterns of mortality during drought dependent on the time since disturbance: R-S+ seedlings survive in greater numbers relative to resprouting species after recent fires, but R-S+ exhibit lower adult survival (higher mortality) in mature stands following longer times since fire.

*Adenostoma fasciculatum* and *A*. *glauca* shrubs with larger crowns had greater survival than smaller ones. This inverse relationship between drought-stress mortality and plant size has also been described for *C*. *megacarpus* [62,63], *A*. *fasciculatum*, *A*. *glauca* and *C*. *greggii* A. Gray [28]. This could be due to a better-established root system or to a greater water storage capacity (capacitance) in larger plants that could alleviate the effects of severe droughts [28,63]. A strategy to cope with drought is adjusting the root to shoot ratio by growing roots or shedding leaf area [64,65]. Larger plants may be better able than smaller plants to increase their root to shoot or root to leaf area ratios through shoot dieback or leaf shedding, thereby increasing their survival. This adjustment could not be maintained indefinitely, as underground biomass requires carbohydrates to be maintained; therefore, such plants risk mortality by way of carbon limitation [12]. This relationship between size and survival may differ for different biomes and growth forms; for example, in tropical forests, larger trees and lianas showed greater mortality than smaller plants during experimental drought [66,67]. Larger common and single-leaf pinyon pine (*Pinus edulis* Engelmann and *Pinus monophylla* Torrey and Fremont, respectively) also show greater mortality than smaller trees in the southwestern United States [68,69]. This may be due to differences in cavitation resistance across tree age, maturity, and size or to bark beetles preferring larger trees [68].

The higher survival of permanently tagged *A*. *fasciculatum* plants in comparison to those randomly selected by PQS was probably related to the size effect previously described. *A*. *fasciculatum* permanently tagged plants were 2.20 ± 0.19 m tall (mean ± SE, n = 10) and the PQS plants were 1.65 ± 0.10 m (n = 86). This height difference was significant (two-sided t-test, P = 0.019). The most likely explanation for this size difference is that all tagged plants were resprouts after the 1993 fire whereas the PQS plants included both resprouts and seedlings that emerged after the fire, the latter presumably having a less extensive root system and a smaller crown. However, for *A*. *sparsifolium*, a species for which the best fit GLM did not detect a size effect on mortality (it is to be noted that only two PQS *A*. *sparsifolium* were dead), the difference in mortality between permanent tagged and PQS plants was smaller (7.5%) and the shrubs of both groups were equal in size (tagged plants height: 3.98 ± 0.14 m (n = 22); PQS plants height: 3.87 ± 0.19 m (n = 23); two side t-test: P = 0.66).

Some hydraulic parameters were correlated with mortality when analysed as species level averages (*i*.*e*. hydraulic parameters were not measured on individuals that later died over the course of the experiment). The Ψ measurements we sampled were lower than those reported for the same species in previous studies (*e*.*g*., [38,48]), which is indicative of the extreme levels of dehydration experienced by plants. Both Ψ_pd_ and Ψ_md_ were correlated with mortality (Fig 7A and 7E) as previously observed with other woody species (*e*.*g*., [7]). All species except *A*. *sparsifolium* showed elevated levels of hydraulic dysfunction (PLC > 60%), but surprisingly PLC did not correlate with mortality despite six of the species showing mean PLC values of 75–90 (Fig 8B), which were in the range of the 88 PLC threshold for mortality described for angiosperms [70]. This may be because during chronic drought stress conduits became permanently blocked and we were unable to flush them back to a representative maximum, as is sometimes the case following embolism [71]. This is consistent with K_max_ values of some of the studied species being lower than those previously reported in this area [38,48]. This indicates a physiological change (*i*.*e*. reduction in conductive vessels) in these drought stressed plants that is not well understood. It may also be that plants die rapidly once PLC reaches values above the 88% threshold and that we were not capable to capture these values with our sampling. In contrast, K_s_ did correlate with mortality. There was a K_s_ threshold (about 0.2) below which physiological function of the species may not be sustained leading to mortality (Fig 8C). The predictive power of K_s_ highlights the importance of analysing hydraulic function in absolute terms (K_s_) and that in some cases expressing data only in relative terms (PLC) does not provide a complete picture [71–73]. Plants do not respond to PLC *per se*, and it is the supply of water to the leaves to replace transpired water that is key.

Chlorophyll fluorescence allows the assessment of how dehydration of leaf tissues affects the photosynthetic efficiency of energy conversion in leaves [74]. During the peak of the 2014 drought, F_v_/F_m_ was highly variable among our 11 sampled species and all were below the optimum range of 0.80–0.83 for C3 plants [46] indicating varying degrees of photoinhibition [74]. Photoinhibition has previously been shown to be heightened in dehydrated Mediterranean plants (*e*.*g*., [22,25,75–77]) and to correlate with survival in seedlings submitted to experimental drought [22]. The strong inverse correlation we found between F_v_/F_m_ and mortality (even stronger than K_s_, Ψ_pd_ and Ψ_md_) suggests that the species with enhanced ability to avoid photoinhibition were more likely to avoid mortality. The strength of this relationship may result from our plants experiencing a climate-change type drought and that a combination of tissue dehydration and high temperatures strained PSII. It is also likely that an increase in the osmotic potential in the cytosol of cells, at water potentials approaching -14 MPa, directly impaired chloroplast function. This is an important finding because chlorophyll fluorescence is an easy, fast, and non-invasive measurement, thus it could be used to evaluate strain levels imposed by intense drought for many species in diverse plant communities providing a valuable predictor of which species are most threatened. This could be of considerable benefit for land managers in deciding when to intervene to rescue rare species or communities. Recent developments in sun-induced chlorophyll fluorescence [78,79] suggest great potential in remote sensing to evaluate the effects of drought-stress on chaparral communities (*cf*. [55]).

In summary, this study shows that intense drought episodes can alter the composition, frequency and relative dominance of species in diverse chaparral communities due to differential mortality among species. The long term impacts of these changes will depend, in part, on future recruitment and disturbance patterns at this site, but they, at minimum, represent a significant short term and rapid change at the site. Hydraulic traits, plant size, and life history type were related to differential drought mortality. Tissue dehydration tolerance does not increase drought-survival *per se*, and, in this study, species with a dehydration avoidance strategy typically showed lower mortality levels. F_v_/F_m_ was the parameter that most strongly correlated with levels of mortality for a species suggesting that photoinhibition of PSII may be a useful measure of stress and mortality risk during climate-change type droughts. F_v_/F_m_ has promise for evaluating chaparral drought induced mortality at a landscape scale.

## Supporting Information

S1 Appendix Formulas used for calculating the point quarter sampling (PQS) parameters. (PDF)

S1 Fig Walter-Lieth climate diagram of the study site. (PDF)

S2 Fig Monthly precipitation (mm) and temperature (°C) data of three weather stations situated closest to the study site for August 2011 –July 2015. (PDF)

S1 Data Spreadsheet containing the data set used to reach the conclusions of this study and the values used for building the figures. (XLSX)

S1 Table Evaluated generalized linear models (GLMs) describing plant mortality. (PDF)

S2 Table Parameter estimates of the best fitting mortality generalized linear model (GLM). (PDF)

## References

[1] Parker VT, Pratt RB, Keeley JE. Chaparral In: Mooney H, Zavaleta E, editors. Terrestrial ecosystems of California. Oakland: University of California Press; 2016 pp. 479–507.

[2] Rice KJ, Matzner SL, Byer W, Brown JR. Patterns of tree dieback in Queensland, Australia: the importance of drought stress and the role of resistance to cavitation. Oecologia. 2004; 139: 190–198. 14767754 10.1007/s00442-004-1503-9

[3] Breshears DD, Cobb NS, Rich PM, Price KP, Allen CD, Balice RG, et al Regional vegetation die-off in response to global-change-type drought. Proc Natl Acad Sci U S A. 2005; 102: 15144–15148. 16217022 10.1073/pnas.0505734102 PMC1250231

[4] van Mantgem PJ, Stephenson NL (2007) Apparent climatically induced increase of tree mortality rates in a temperate forest. Ecol Lett. 2007; 10: 909–916. 17845291 10.1111/j.1461-0248.2007.01080.x

[5] Allen CD, Macalady AK, Chenchouni H, Bachelet D, McDowell N, Vennetier M, et al A global overview of drought and heat-induced tree mortality reveals emerging climate change risks for forests. For Ecol Manage. 2010; 259: 660–684.

[6] Anderegg WRL, Berry JA, Smith DD, Sperry JS, Anderegg LDL, Field CB. The roles of hydraulic and carbon stress in a widespread climate-induced forest die-off. Proc Natl Acad Sci U S A. 2012; 109: 233–237. 10.1073/pnas.1107891109 22167807 PMC3252909

[7] McDowell NG, Williams AP, Xu C, Pockman WT, Dickman LT, Sevanto S, et al Multi-scale predictions of massive conifer mortality due to chronic temperature rise. Nat Clim Chang. 2016; 6: 295–300.

[8] M.E.A. Board. Ecosystems and human well-being: synthesis Millennium Ecosystem Assessment Board. Washington, DC: Island Press; 2005.

[9] Moser S, Ekstrom J, Franco G. Our changing climate 2012. Vulnerability and adaptation to the increasing risks from climate change in California. Summary Brochure, Report on the Third Assessment from the California Climate Change Center, Sacramento, CA; 2012.

[10] IPCC. Climate change 2014: synthesis report. Contribution of working groups I, II and III to the fifth assessment report of the Intergovernmental Panel on Climate Change [Core Writing Team, R.K. Pachauri and L.A. Meyer (eds.)]. IPCC, Geneva, Switzerland; 2014.

[11] Cook BI, Ault TR, Smerdon JE. Unprecedented 21st century drought risk in the American Southwest and Central Plains. Sci Adv. 2015; 1: e1400082 10.1126/sciadv.1400082 26601131 PMC4644081

[12] McDowell NG, Pockman WT, Allen CD, Breshears DD, Cobb N, Kolb T, et al Mechanisms of plant survival and mortality during drought: why do some plants survive while others succumb to drought? New Phytol. 2008; 178: 719–739. 10.1111/j.1469-8137.2008.02436.x 18422905

[13] Anderegg WRL, Plavcová L, Anderegg LDL, Hacke UG, Berry JA, Field CB. Drought's legacy: multiyear hydraulic deterioration underlies widespread aspen forest die‐off and portends increased future risk. Glob Chang Biol. 2013; 19: 1188–1196. 10.1111/gcb.12100 23504895

[14] Plaut JA, Yepez EA, Hill J, Pangle R, Sperry JS, Pockman WT, McDowell NG. Hydraulic limits preceding mortality in a piñon–juniper woodland under experimental drought. Plant Cell Environ. 2012; 35: 1601–1617. 10.1111/j.1365-3040.2012.02512.x 22462824

[15] Oliva J, Stenlid J, Martinez-Vilalta J. The effect of fungal pathogens on the water and carbon economy of trees: implications for drought-induced mortality. New Phytol. 2014; 203: 1028–1035. 10.1111/nph.12857 24824859

[16] Sala A, Piper F, Hoch G. Physiological mechanisms of drought-induced tree mortality are far from being resolved. New Phytol. 2010; 186: 274–281. 10.1111/j.1469-8137.2009.03167.x 20409184

[17] Körner C. Paradigm shift in plant growth control. Curr Opin Plant Biol. 2015; 25:107–114. 10.1016/j.pbi.2015.05.003 26037389

[18] McDowell NG, Beerling DJ, Breshears DD, Fisher RA, Raffa KF, Stitt M. The interdependence of mechanisms underlying climate-driven vegetation mortality. Trends Ecol Evol. 2011; 26: 523–532. 10.1016/j.tree.2011.06.003 21802765

[19] Sevanto S, McDowell NG, Dickman LT, Pangle R, Pockman WT. How do trees die? A test of the hydraulic failure and carbon starvation hypotheses. Plant Cell Environ. 2014; 37: 153–161. 10.1111/pce.12141 23730972 PMC4280888

[20] Mencuccini M, Minunno F, Salmon Y, Martínez-Vilalta J, Hölttä T. Coordination of physiological traits involved in drought-induced mortality of woody plants. New Phytol. 2015; 208: 396–409. 10.1111/nph.13461 25988920

[21] Salmon Y, Torres-Ruiz JM, Poyatos R, Martínez-Vilalta J, Meir P, Cochard H, et al Balancing the risks of hydraulic failure and carbon starvation: a twig scale analysis in declining Scots pine. Plant Cell Environ. 2015; 38: 2575–2588. 10.1111/pce.12572 25997464 PMC4989476

[22] Valladares F, Sánchez-Gómez D. Ecophysiological traits associated with drought in Mediterranean tree seedlings: individual responses versus interspecific trends in eleven species. Plant Biol. 2006; 8: 688–697. 16773558 10.1055/s-2006-924107

[23] Pratt RB, Jacobsen AL, Mohla R, Ewers FW, Davis SD. Linkage between water stress tolerance and life history type in seedlings of nine chaparral species (Rhamnaceae). J Ecol. 2008; 96: 1252–1265.

[24] Pratt RB, Jacobsen AL, Ramirez AR, Helms AM, Traugh CA, Tobin MF, et al Mortality of resprouting chaparral shrubs after a fire and during a record drought: physiological mechanisms and demographic consequences. Glob Chang Biol. 2014; 20: 893–907. 10.1111/gcb.12477 24375846

[25] Vilagrosa A, Morales F, Abadía A, Bellot J, Cochard H, Gil-Pelegrin E. Are symplast tolerance to intense drought conditions and xylem vulnerability to cavitation coordinated? An integrated analysis of photosynthetic, hydraulic and leaf level processes in two Mediterranean drought-resistant species. Environ Exp Bot. 2010; 69: 233–242.

[26] Skelton RP, West AG, Dawson TE. Predicting plant vulnerability to drought in biodiverse regions using functional traits. Proc Natl Acad Sci U S A. 2015; 112: 5744–5749. 10.1073/pnas.1503376112 25902534 PMC4426410

[27] Davis SD, Ewers FW, Sperry JS, Portwood KA, Crocker MC, Adams GC. Shoot dieback during prolonged drought in *Ceanothus* (Rhamnaceae) chaparral of California: a passible case of hydraulic failure. Am J Bot. 2002; 89: 820–828. 10.3732/ajb.89.5.820 21665682

[28] Paddock WAS III, Davis SD, Pratt RB, Jacobsen AL, Tobin MF, Lopez-Portillo J, Ewers FW. Factors determining mortality of adult chaparral shrubs in an extreme drought year in California. Aliso. 2013; 31: 49–57.

[29] Griffin D, Anchukaitis KJ. How unusual is the 2012–2014 California drought? Geophys Res Lett. 2014; 41: 9017–9023.

[30] Mann ME, Gleick PH. Climate change and California drought in the 21st century. Proc Natl Acad Sci U S A. 2015; 112: 3858–3859. 10.1073/pnas.1503667112 25829537 PMC4386383

[31] Pausas JG, Pratt RB, Keeley JE, Jacobsen AL, Ramirez AR, Vilagrosa A, et al Towards understanding resprouting at the global scale. New Phytol. 2016; 209: 945–954. 10.1111/nph.13644 26443127

[32] Bond WJ, Midgley JJ. Ecology of sprouting in woody plants: the persistence niche. Trends Ecol Evol. 2001; 16: 45–51. 11146144 10.1016/s0169-5347(00)02033-4

[33] Pratt RB, Jacobsen AL, Hernandez J, Ewers FW, North GB, Davis SD. Allocation tradeoffs among chaparral shrub seedlings with different life history types (Rhamnaceae). Am J Bot. 2012; 99: 1464–1476. 10.3732/ajb.1200193 22917948

[34] Swain DL, Horton DE, Singh D, Diffenbaugh NS. Trends in atmospheric patterns conducive to seasonal precipitation and temperature extremes in California. 2016; Science Advances. 2: e1501344 10.1126/sciadv.1501344 27051876 PMC4820386

[35] Barton K. Re-establishment of *Adenostoma fasciculatum* and *A* *sparsifolium* chaparral after the Malibu wildfire of 1993. Honours Thesis, Pepperdine University. 1995.

[36] Keeley JE, Fotheringham CJ. Historic fire regime in southern California shrublands. Conserv Biol. 2001; 15: 1536–1548.

[37] Witter M, Taylor RS, Davis S. Vegetation response to wildfire and fire history in the Santa Monica Mountains In: Knapp DA, editor. Flora and ecology of the Santa Monica Mountains. Fullerton: Southern California Botanists; 2007 pp. 173–194.

[38] Jacobsen AL, Pratt RB, Ewers FW, Davis SD. Cavitation resistance among 26 chaparral species of southern California. Ecol Monogr. 2007; 77: 99–115.

[39] Hacke UG, Jacobsen AL, Pratt RB. Xylem function of arid-land shrubs from California, USA: an ecological and evolutionary analysis. Plant Cell Environ. 2009; 32: 1324–1333. 10.1111/j.1365-3040.2009.02000.x 19453480

[40] Cottam G, Curtis JT, Hale BW. Some sampling characteristics of a population of randomly dispersed individuals. Ecology. 1953; 34: 741–757.

[41] Cox G. Laboratory manual of general ecology Fifth edition Dubuque, Iowa, USA: W. C. Brown; 1985.

[42] Wiens D, Allphin L, Wall M, Slaton MR, Davis SD. Population decline in *Adenostoma sparsifolium* (Rosaceae): an ecogenetic hypothesis for background extinction. Biol J Linn Soc Lond. 2012; 105: 269–292.

[43] Venturas MD, MacKinnon ED, Jacobsen AL, Pratt RB. Excising stem samples under water at native tension does not induce xylem cavitation. Plant Cell Environ. 2015; 38: 1060–1068. 10.1111/pce.12461 25292257

[44] Sperry JS, Donnelly JR, Tyree MT. A method for measuring hydraulic conductivity and embolism in xylem. Plant Cell Environ. 1988; 11: 35–40.

[45] Hacke UG, Sperry JS, Pittermann J. Drought experience and cavitation resistance in six shrubs from the Great Basin, Utah. Basic Appl Ecol. 2000; 1: 31–41.

[46] Björkman O, Demmig B. Photon yield of O_2_ evolution and chlorophyll fluorescence characteristics at 77 K among vascular plants of diverse origins. Planta. 1987; 170: 489–504. 10.1007/BF00402983 24233012

[47] R Core Team. R: A language and environment for statistical computing R Foundation for Statistical Computing, Vienna, Austria 2013 URL http://www.R-project.org

[48] Jacobsen AL, Pratt RB, Davis SD, Ewers FW. Cavitation resistance and seasonal hydraulics differ among three arid Californian plant communities. Plant Cell Envir. 2007; 30: 1599–1609. 10.1111/j.1365-3040.2007.01729.x 17927695

[49] Davis SD, Ewers FW, Wood J, Reeves JJ, Kolb KJ. Differential susceptibility to xylem cavitation among three pairs of *Ceanothus* species in the Transverse Mountain Ranges of southern California. Ecoscience. 1999; 6: 180–186.

[50] Ackerly D. Functional strategies of chaparral shrubs in relation to seasonal water deficit and disturbance. Ecol Monogr. 2004; 74: 25–44.

[51] Pratt RB, Jacobsen AL, Golgotiu KA, Sperry JS, Ewers FW, Davis SD. Life history type and water stress tolerance in nine California chaparral species (Rhamnaceae). Ecol Monogr. 2007; 77: 239–253.

[52] Thomas CM, Davis SD. Recovery patterns of three chaparral shrub species after wildfire. Oecologia. 1989; 80: 309–320. 28312058 10.1007/BF00379032

[53] Meentemeyer RK, Moody A, Franklin J. Landscape-scale patterns of shrub-species abundance in California chaparral. Plant Ecol. 2001; 156: 19–41.

[54] Horton JS, Kraebel CJ. Development of vegetation after fire in the chamise chaparral of southern California. Ecology. 1955; 36: 244–262.

[55] Coates AR, Dennison PE, Roberts DA, Roth KL. Monitoring the impacts of severe drought on southern California chaparral species using hyperspectral and thermal infrared imagery. Remote Sensing. 2015; 7: 14276–14291.

[56] Redtfeldt RA, Davis SD. Physiological and morphological evidence of niche segregation between two co-occurring species of *Adenostoma* in California chaparral. Ecoscience. 1996; 3: 290–296.

[57] Hoffmann WA, Marchin RM, Abit P, Lau OL. Hydraulic failure and tree dieback are associated with high wood density in a temperate forest under extreme drought. Glob Chang Biol. 2011; 17: 2731–2742.

[58] Hellmers H, Horton JS, Juhren G, O'Keefe J. Root systems of some chaparral plants in southern California. Ecology. 1955; 36: 667–678.

[59] Padilla FM, Pugnaire FI. Rooting depth and soil moisture control Mediterranean woody seedling survival during drought. Funct Ecol. 2007; 21: 489–495.

[60] Ovalle JF, Arellano EC, Ginocchio R. Trade-offs between drought survival and rooting strategy of two South American Mediterranean tree species: implications for dryland forests restoration. Forests. 2015; 6: 3733–3747.

[61] Frazer JM, Davis SD. Differential survival of chaparral seedlings during the first summer drought after wildfire. Oecologia. 1988; 76: 215–221. 28312199 10.1007/BF00379955

[62] Schlesinger WH, Gill DS. Biomass, production, and changes in the availability of light, water, and nutrients during the development of pure stands of the chaparral shrub, *Ceanothus megacarpus*, after fire. Ecology. 1980; 61: 781–789.

[63] Schlesinger WH, Gray JT, Gill DS, Mahall BE. *Ceanothus megacarpus* chaparral: a synthesis of ecosystem processes during development and annual growth. The Botanical Review. 1982; 48: 71–117.

[64] Maseda PH, Fernández RJ. Stay wet or else: three ways in which plants can adjust hydraulically to their environment. J Exp Bot. 2006; 57: 3963–3977. 17079697 10.1093/jxb/erl127

[65] Miranda JdD Padilla FM, Martínez-Vilalta J Pugnaire FI. Woody species of a semi-arid community are only moderately resistant to cavitation. Funct Plant Biol. 2010; 37: 828–839.

[66] Nepstad DC, Tohver IM, Ray D, Moutinho P, Cardinot G. Mortality of large trees and lianas following experimental drought in an Amazon forest. Ecology. 2007; 88: 2259–2269. 17918404 10.1890/06-1046.1

[67] Rowland L, Da Costa ACL, Galbraith DR, Oliveira RS, Binks OJ, Oliveira AAR, et al Death from drought in tropical forests is triggered by hydraulics not carbon starvation. Nature. 2015; 528: 119–122. 10.1038/nature15539 26595275

[68] Mueller RC, Scudder CM, Porter ME, Trotter RT, Gehring CA, Whitham TG. Differential tree mortality in response to severe drought: evidence for long-term vegetation shifts. J Ecol. 2005; 93: 1085–1093.

[69] Meddens AJH, Hicke JA, Macalady AK, Buotte PC, Cowles TR, Allen CD. Patterns and causes of observed pinon pine mortality in the southwestern United States. New Phytol. 2015; 206: 91–97. 10.1111/nph.13193 25494578

[70] Urli M, Porté AJ, Cochard H, Guengant Y, Burlett R, Delzon S. Xylem embolism threshold for catastrophic hydraulic failure in angiosperm trees. Tree Physiol. 2013; 33: 672–683. 10.1093/treephys/tpt030 23658197

[71] Jacobsen AL, Pratt RB. No evidence for an open vessel effect in centrifuge‐based vulnerability curves of a long‐vesselled liana (*Vitis vinifera*). New Phytol. 2012; 194: 982–990. 10.1111/j.1469-8137.2012.04118.x 22448870

[72] Sperry JS, Christman MA, Torres-Ruiz JM, Taneda H, Smith DD. Vulnerability curves by centrifugation: is there an open vessel artefact, and are ‘r’shaped curves necessarily invalid? Plant Cell Environ. 2012; 35: 601–610. 10.1111/j.1365-3040.2011.02439.x 21988455

[73] Hacke UG, Venturas MD, MacKinnon ED, Jacobsen AL, Sperry JS, Pratt RB. The standard centrifuge method accurately measures vulnerability curves of long‐vesselled olive stems. New Phytol. 2015; 205: 116–127. 10.1111/nph.13017 25229841

[74] Maxwell K, Johnson GN. Chlorophyll fluorescence—a practical guide. J Exp Bot. 2000; 51: 659–668. 10938857 10.1093/jxb/51.345.659

[75] Epron D, Dreyer E, Bréda N. Photosynthesis of oak trees [*Quercus petraea* (Matt.) Liebl.] during drought under field conditions: diurnal course of net CO_2_ assimilation and photochemical efficiency of photosystem II. Plant Cell Environ. 1992; 15: 809–820.

[76] Damesin C, Rambal S. Field study of leaf photosynthetic performance by a Mediterranean deciduous oak tree (*Quercus pubescens*) during a severe summer drought. New Phytol. 1995; 131: 159–167.

[77] Rodríguez-Calcerrada J, Pardos JA, Gil L, Aranda I. Summer field performance of *Quercus petraea* (Matt.) Liebl and *Quercus pyrenaica* Willd seedlings, planted in three sites with contrasting canopy cover. New Forests. 2007; 33: 67–80.

[78] Guanter L, Zhang Y, Jung M, Joiner J, Voigt M, Berry JA, et al Global and time-resolved monitoring of crop photosynthesis with chlorophyll fluorescence. Proc Natl Acad Sci U S A. 2014; 111: E1327–E1333. 10.1073/pnas.1320008111 24706867 PMC3986187

[79] Porcar-Castell A, Tyystjarvi E, Atherton A, van der Tol C, Flexas J, Pfündel EE, et al Linking chlorophyll a fluorescence to photosynthesis for remote sensing application: mechanism and challenges. J Exp Bot. 2014; 65: 4065–4095. 10.1093/jxb/eru191 24868038

