# Supplementary material for: Chaparral Shrub Hydraulic Traits, Size, and Life History Types Relate to Species Mortality during California’s Historic Drought of 2014
Source: PLoS One. 2016 Jul 8;11(7):e0159145. doi: 10.1371/journal.pone.0159145 (PMC4938587; doi:10.1371/journal.pone.0159145)
Supplement: S1 Appendix — (PDF) [file pone.0159145.s001.pdf]

S1 Appendix. Formulas used for calculating the point quarter sampling (PQS) parameters (Cox, 1985).

Parameters calculated for the whole stand:

$$Total\ density\ of\ all\ species\ (plants\ ha^{-1}) = \frac{10{,}000}{(mean\ point\ to\ plant\ distance\ in\ meters)^2}$$

Parameters calculated for each species:

$$Relative\ density = \frac{individuals\ of\ a\ species}{total\ individuals\ of\ all\ species} \times 100$$

$$Density = \frac{relative\ density\ of\ a\ species}{100} \times total\ density\ of\ all\ species$$

$$Dominance = density\ of\ a\ species\ \times\ average\ crown\ area\ \ of\ the\ plants\ of\ each\ species$$

(Dominance can also be calculated with the average basal area of each species)

$$Relative\ dominance = \frac{dominance\ for\ a\ species}{total\ dominance\ for\ all\ species} \times 100$$

$$Frequency = \frac{number\ of\ points\ at\ which\ species\ occurs}{total\ number\ of\ points\ sampled}$$

$$Relative\ frequency = \frac{frequency\ value\ for\ a\ species}{total\ of\ frequency\ values\ for\ all\ species} \times 100$$

$$Importance\ value = relative\ density + relative\ dominance + relative\ frequency$$

Reference: Cox G (1985) Laboratory manual of general ecology. Fifth edition. W. C. Brown, Dubuque, Iowa, USA.
