# Supplementary material for: Chaparral Shrub Hydraulic Traits, Size, and Life History Types Relate to Species Mortality during California’s Historic Drought of 2014
Source: PLoS One. 2016 Jul 8;11(7):e0159145. doi: 10.1371/journal.pone.0159145 (PMC4938587; doi:10.1371/journal.pone.0159145)
Supplement: S1 Fig — (PDF) [file pone.0159145.s002.pdf]

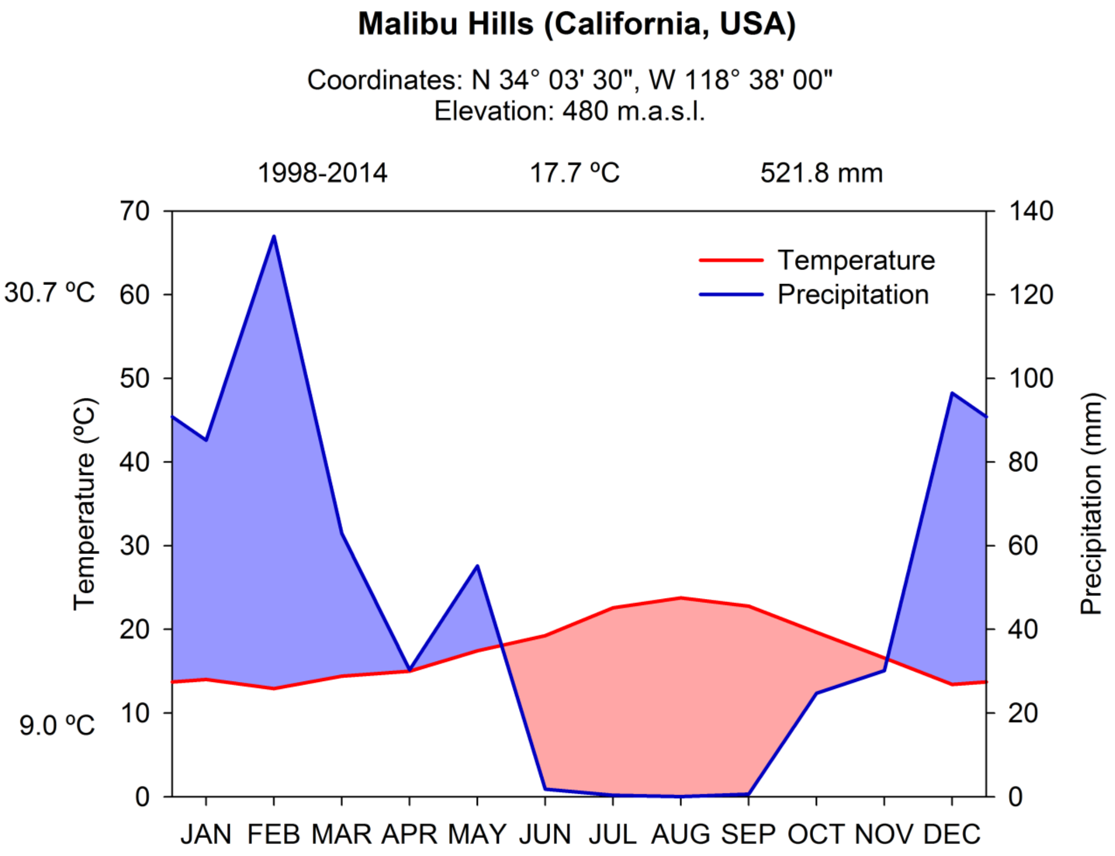

S1 Figure. Walter-Lieth climate diagram of the study site. Data obtained from Malibu Hills climate station as it is the one with the longest records out of the three closest to the study site (USDA Forest Service). The information above the panel corresponds to station location, the period of years recorded, the mean annual temperature and the mean annual precipitation. Data situated top-left from temperature axis corresponds to the mean of the average daily maximum temperature of the hottest month and bottom left from the same axis the mean of the average daily minimum temperature of the coldest month. Area shaded in blue indicates the moist period and shaded in red the arid period.
