# Supplementary material for: Chaparral Shrub Hydraulic Traits, Size, and Life History Types Relate to Species Mortality during California’s Historic Drought of 2014
Source: PLoS One. 2016 Jul 8;11(7):e0159145. doi: 10.1371/journal.pone.0159145 (PMC4938587; doi:10.1371/journal.pone.0159145)
Supplement: S2 Fig — (PDF) [file pone.0159145.s003.pdf]

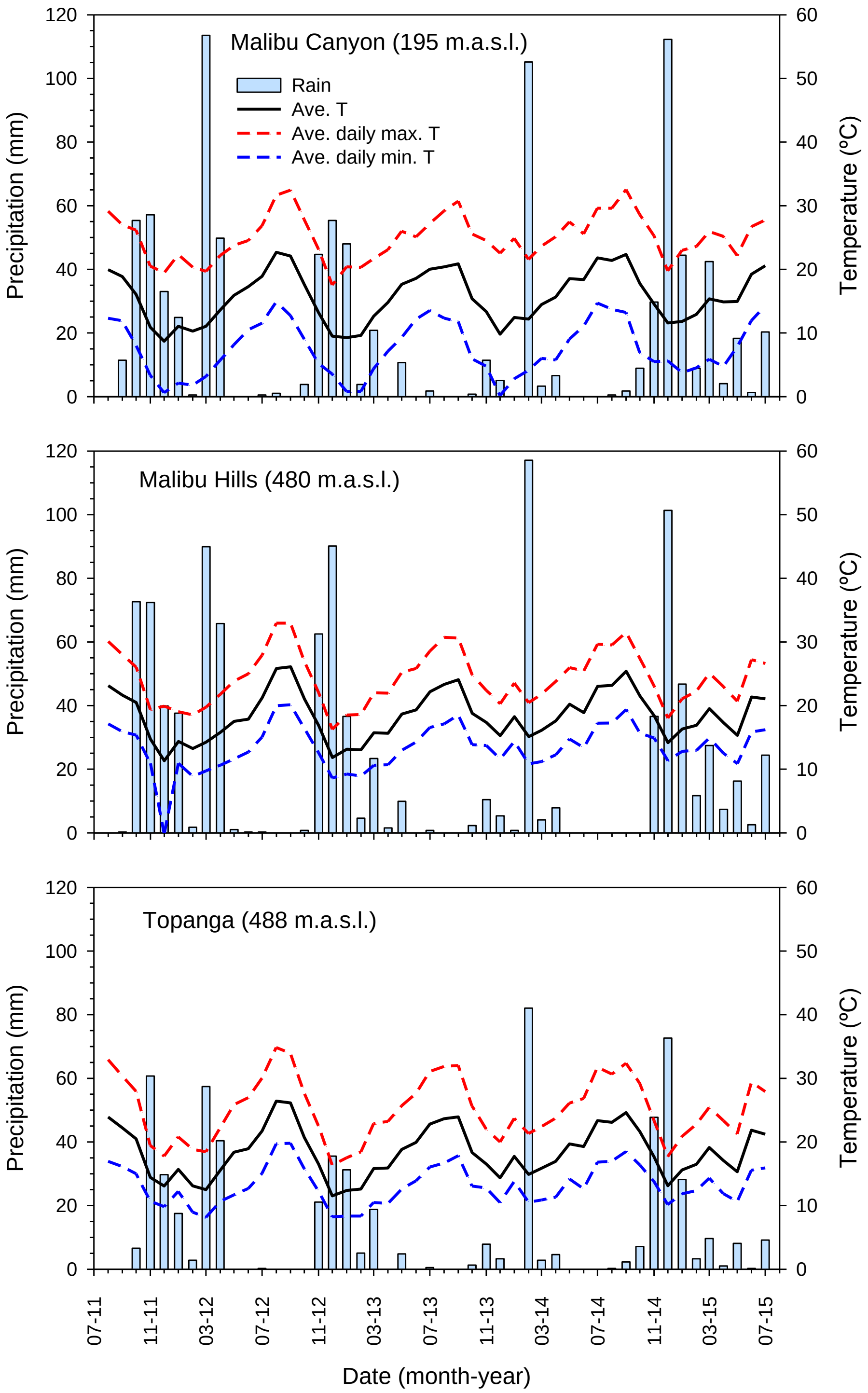

S2 Figure. Monthly precipitation (mm) and temperature (ºC) data of three weather stations situated close to the study site: Malibu Canyon (N 34º 05' 59'', W 118º 42' 12''; distance to the study site: 5.00 km; NESS ID: CA29660E), Malibu Hills (N 34º 03' 30'', W 118º 38' 00''; distance to the study site: 3.86 km; NESS ID: CA4173E), and Topanga (N 34º 08' 10'', W 118º 36' 22''; distance to the study site: 6.54 km; NESS ID: 2E9123E4). The weather stations belong to the State and Private Forestry organization of the USDA Forest Service. Data from August 2011 to July 2015 (4 years).
