# Supplementary material for: Chaparral Shrub Hydraulic Traits, Size, and Life History Types Relate to Species Mortality during California’s Historic Drought of 2014
Source: PLoS One. 2016 Jul 8;11(7):e0159145. doi: 10.1371/journal.pone.0159145 (PMC4938587; doi:10.1371/journal.pone.0159145)
Supplement: S1 Table — (PDF) [file pone.0159145.s005.pdf]

S1 Table. Generalized linear models (GLMs) describing plant mortality. The model that best describes mortality is marked in bold, *i.e.* the one with the lowest Akaike Information Criterion (AIC).

Factors[†]	Prob>ChiSq	AIC
Sp, H, BA, CA, D	< 0.0001	275.7
H, BA, CA, D	< 0.0001	278.1
Sp, BA, CA, D	< 0.0001	273.7
Sp, H, CA, D	< 0.0001	273.6
Sp, H, BA, D	< 0.0001	286.8
Sp, H, BA, CA	< 0.0001	274.0
Sp, H, BA	< 0.0001	286.1
Sp, BA, CA	< 0.0001	272.0
Sp, CA, D	< 0.0001	271.6
Sp, H, CA	< 0.0001	271.9
Sp, H, D	< 0.0001	285.1
Sp, BA, D	< 0.0001	287.9
H, BA, CA	< 0.0001	276.6
H, CA, D	< 0.0001	276.7
H, BA, D	< 0.0001	303.0
BA, CA, D	< 0.0001	276.6
Sp, H	< 0.0001	284.6
Sp, BA	< 0.0001	287.5
Sp, CA	< 0.0001	269.9
Sp, D	< 0.0001	286.3
H, BA	< 0.0001	302.7
H, CA	< 0.0001	275.3
H, D	0.0007	309.3
BA, CA	< 0.0001	275.2
BA, D	< 0.0001	305.4
CA, D	< 0.0001	275.3
Sp	< 0.0001	286.2
H	0.0005	309.9
BA	< 0.0001	305.9
CA	< 0.0001	273.9
D	0.0297	317.2
Sp, CA, Sp×CA[‡]	< 0.0001	262.2
**Sp, CA(Sp)**[§]	< **0.0001**	**260.0**
Sp, BA(Sp)[§]	< 0.0001	284.2
Sp, H(Sp)[§]	< 0.0001	273.4

[†] Factor codes: species, Sp; plant height, H; basal area, BA; crown area, CA; local density, D.

[‡] Interaction between factors.

[§] Nested factor.
