# Supplementary material for: Chaparral Shrub Hydraulic Traits, Size, and Life History Types Relate to Species Mortality during California’s Historic Drought of 2014
Source: PLoS One. 2016 Jul 8;11(7):e0159145. doi: 10.1371/journal.pone.0159145 (PMC4938587; doi:10.1371/journal.pone.0159145)
Supplement: S2 Table — (PDF) [file pone.0159145.s006.pdf]

S2 Table. Parameter estimates of the best fit mortality GLM. Significant parameters are marked in bold.

Term	Estimate	Standard Error	L-R Chi Square	Prob. > Chi Square
Intercept	0.63	0.39	3.4	0.0666
Sp[Af]	-0.34	0.43	0.7	0.4019
Sp[Ag]	-0.12	0.56	0.0	0.8289
**Sp[As]**	-1.93	0.84	6.4	**0.0117**
Sp[Cc]	0.29	0.44	0.4	0.5497
**Sp[Af]:(CA-2.31663)**	-0.39	0.12	13.2	**0.0003**
**Sp[Ag]:(CA-2.31663)**	-1.17	0.38	19.6	**< 0.0001**
Sp[As]:(CA-2.31663)	-0.21	0.18	1.8	0.185
Sp[Cc]:(CA-2.31663)	0.00	0.13	0.0	0.9732
Sp[Cs]:(CA-2.31663)	0.35	0.96	0.2	0.6696
